# Comprehending scientific metaphors in the bilingual brain: Evidence from event-related potentials

**DOI:** 10.3389/fpsyg.2022.1037525

**Published:** 2022-12-06

**Authors:** Lexian Shen, Xiaoguang Li, Shaojuan Huang, Yanhong Huang, Xinyu Gao, Ziqing You, Zirun Mao, Xuemei Tang

**Affiliations:** ^1^School of Foreign Studies, Anhui Polytechnic University, Wuhu, China; ^2^Jinling High School, Nanjing, China

**Keywords:** scientific metaphors, bilingual, event-related potentials, N400, late negativity

## Abstract

While the processing mechanisms of novel and conventional metaphors were widely investigated in previous monolingual studies, little attention has been devoted to how metaphoric utterances are processed by the bilingual brain as well as how scientific context might modulate such processes. Using event-related potentials (ERPs), this paper investigates the way in which scientific metaphors are electrophysiologically processed in Chinese (L1) and English (L2), with the aim of investigating the different mechanisms for understanding metaphorical language in first (L1) and second (L2) languages. By time-locking the N400 and later LPC time windows, the research show how meaning integration differs between L1 and L2 at different stages when comprehending figurative language. We found that compared with Chinese scientific metaphors, English scientific metaphors elicited greater N400, smaller late positive component (LPC), and greater late negativity, and English literals elicited greater late negativity. Our findings suggest that the dynamics of processing figurative meaning in bilingual brains over time show a complex pattern, with language, context, inference and salience jointly modulating temporal dynamics and possible cerebral asymmetries, supporting the revised hierarchical model.

## Introduction

Metaphors are often used in the scientific field to talk about a less familiar domain (e.g., *lymph*) with a more familiar domain (e.g., *police*). In the LYMPH IS POLICE metaphor, the systematic function parallelisms of patrolling (in our body and in a place) is formed across languages. Such linguistic pattern suggests that the use of concrete terms is quite practical to describe abstract even intangible scientific concepts.

According to previous studies (Tang et al., 2017a,b), the target and source domains in scientific metaphors (e.g., *The lymph is a policeman*) are from different contexts, namely, the scientific target (*lymph*) and the daily source (*policeman*). Consequently, retrieving the stored conceptual knowledge associated with scientific metaphors is much more demanding than retrieving knowledge of literal expressions. Besides, the processing of scientific metaphors might also involve a secondary integration of meaning compared with literal expressions.

Moreover, the processing of scientific metaphors may also involve a later conceptual mapping according to some psycholinguistic models. The structural-mapping model (Gentner, 1983) and the career of a metaphor model (Gentner and Wolff, 1997; Bowdle and Gentner, 2005) suggested the mapping process involves comparing similarities between the source and the target. Accordingly, with more complicated contexts and deeper integration for analogical inference, compared with conventional metaphors (e.g., *Books are friends*), scientific metaphors should show more clearly the slower and more difficult mapping process of metaphors due to the absence of well-defined associations between the two domains. For example, in “*The intervening lymph nodes can trap and destroy the cancer cells.*,” the target concept LYMPH and the source concept POLICE are aligned by the predicate “*trap and destroy*,” and therefore, the role played by the lymphatic system in the body’s immune system “*lymph*” is described as the role played by the political system in a place.

Then, what is the basic mechanism underlying understanding scientific metaphors in L2 (second language) for late learners, and to what extent do they overlap with the mechanisms involved in scientific metaphor processing in L1 (first language)? According to the non-selective access/integrated view, words in the L2 should behave like low-frequency words in the L1 (Dijkstra et al., 1998; Lemhofer et al., 2008). However, according to the revised hierarchical model (Kroll and Stewart, 1994), it would be an unlikely possibility for late learners of L2 to process words in the similar way L1 learners do, and the mechanisms for processing words in L2 and L1 are quite different. At least, anecdotally, many L2 learners report using translation into L1 as a general heuristic for processing L2 words (van Der Meij et al., 2011), resulting in the switch cost (slower response latencies; Verhoef et al., 2009; Calabria et al., 2011).

Actually, the comparison between performance in L2 and that in L1 might be modulated by the frequency characteristics of the words in each language (Midgley et al., 2009). Exposure to L1 words is generally much higher than exposure to L2 words. Therefore, the subjective frequencies of L2 words might be lower than those of L1 words, which could be driving the behavioral effects. Furthermore, age-of-acquisition could be another factor (Wang and Chen, 2020), and L2 words are learned later than the majority of L1 words. Finally, the lexical-semantic connection between L2 words is much weaker than that between L1 words (Midgley et al., 2009; Naranowicz et al., 2022). In order to observe the modulation effects of the factors mentioned above, the current study adopted scientific metaphors as the target stimuli. Scientific terms used in scientific metaphors distinguish them from other kinds of metaphors. Generally speaking, either in L1 or L2, exposure to scientific words is lower than exposure to daily words; they are used and learned later than daily words; the interconnection between scientific words and daily words are weaker than that between different daily words.

The aim of the present study was to examine the neural mechanisms for bilingual speakers (L1 Chinese and L2 English) to process figurative language with scientific metaphors as the stimuli. Adopting scientific metaphors with complicated contextual structure and knowledge-inferencing process might be of more significance to show the difference between the processing of L1 and L2.

### Models for metaphor processing

According to the graded salience hypothesis (Giora, 2003), the time-course of meaning processing is mainly determined by the degree of meaning salience which refers to those meanings foremost in speakers’ minds at time of speaking characterized by conventionality, prototypicality, familiarity, and frequency. The meaning of literal expressions is commonly salient, and is processed first in the left hemisphere. In contrast, for a novel or unfamiliar metaphor, the salient meaning is the literal one, and the figurative meaning is inferred later by contextual mechanisms and is processed mainly in the right hemisphere. In other words, how linguistic stimuli are processed is determined by the salience-non-salience continuum instead of the literal metaphoric distinction. This hypothesis predicts a later and right-hemisphere biased processing of nonsalient meanings (such as scientific metaphors in L1) and an earlier and left-hemisphere biased processing of salient meanings (such as literal expressions in L1).

The Fine-Coarse Semantic Coding Theory (Beeman, 1998, 2005) is another psycholinguistic theory that addresses hemispheric functions in semantic processing, which proposed that language is processed qualitatively differently by the two cerebral hemispheres. The right hemisphere loosely activates and maintains larger semantic fields containing more distant associates and more unconventional meanings (coarse semantic coding) whereas the left hemisphere focuses on a single dominant interpretation (fine semantic coding). Since the distance between the source and target domains is usually semantically longer than that for literal expressions, the theory predicts semantic processes in the right hemisphere may be more apt for scientific metaphor comprehension in L1.

More importantly, these two models might have some important implications when comparing L1 and L2 metaphor processing. For native speakers, a literal expression possesses a highly salient interpretation since native speakers have naturally encountered the expressions quite often in their daily life. Thus, on encountering a highly conventional expression, the left hemisphere engages in a fine coding and strong activation of small semantic fields. And for scientific metaphors, it might be of little difficulty for L1 speakers to understand the literal meaning of scientific metaphors. It might be difficult for them to achieve the mapping between the distant domains covering two different contexts and to make scientific inference to get some sort of new knowledge, probably involving a coarse processing of meanings in the right hemisphere.

However, for later learners of L2, the picture is more complicated. At the early stage of processing the meaning, the literal meaning of scientific metaphors may be less salient, resulting in relatively longer period of processing and stronger activation of the right hemisphere of coarse semantic coding. At the later stage of mapping and inference, it might be very difficult to deeply integrate the meaning in L2, probably resulting in delayed reintegration of meaning, stronger activation of the left hemisphere of fine semantic coding and the weaker activation of large and diffuse semantic fields in the right hemisphere.

### Event-related potentials (ERPs) and bilingual metaphor processing

The mean amplitudes of N400 were widely investigated to reveal the processing mechanism of metaphors. Previous studies show that N400 amplitudes are sensitive to semantic violations (Kutas and Hillyard, 1980) and are modulated by several variables on the mechanism of figurative language processing, such as familiarity, difficulty, context or task (Schmidt and Seger, 2009). Meanwhile, some bilingual ERP studies reported a modulation of N400 amplitudes by language (Newman et al., 2012; Heidlmayr et al., 2015), with L2 eliciting lower N400 compared with L1.

The late positive component (LPC) is considered to reflect integration or reprocessing at the sentence level (Kaan et al., 2000) and is found to be modulated by the degree of conventionality (Weiland et al., 2014) with novel metaphors eliciting greater LPCs than conventional metaphors and literal expressions. Other studies reported a late negativity overlapping in the LPC time window resulting in smaller LPCs elicited by novel metaphors compared with conventional metaphors indicating a sustained difficulty in fusing two concepts of the source domain and the target domain (Arzouan et al., 2007a,b; Goldstein et al., 2012; Rutter et al., 2012).

The feasibility of investigating the processing mechanism of scientific metaphors using amplitudes of N400 and late negativity was confirmed in our previous monolingual studies (Tang et al., 2017a,b). Compared with conventional metaphors and literal expressions, scientific metaphors and poetic metaphors elicited higher N400s reflecting the modulation of conventionality. More importantly, scientific metaphors elicited larger late negativity compared with poetic metaphors and conventional metaphors (Tang et al., 2017b).

Moreover, for the bilingual studies, N400 peak latency was also proved to be susceptible to biological and cognitive influences (Moreno et al., 2008). For example, a delay in N400 latency is associated with increasing the age of participants and increasing stimulus presentation rate (Midgley et al., 2009). Most of the studies examined the N400 event-related response with a semantic violation paradigm and reported a significant delay in its peak latency for L2 compared with L1 (Hahne and Friederici, 2001; Moreno and Kutas, 2005). For example, the peak latency of the N400 effect elicited by non-dominant language target word was significantly later (approximately 27 ms) than that elicited by dominant language target words (Moreno and Kutas, 2005). It appears that both the age of exposure to the language and the proficiency of the language are factors in the delay of the peak latency of the N400 response to semantic incongruities. However, the factors contributing to delays in the N400 response in bilinguals in their L2 vs. L1 demands further investigation because an early age of exposure does not always guarantee a fast response to semantic incongruity. Other modulations of N400 parameters such as amplitude, onset latency, and scalp distribution are not consistent across studies (Anna and Roberto, 2011; Mashal et al., 2015).

### The present study

While much research has been done on novel and conventional metaphor comprehension in the monolingual context, little attention has been devoted to how metaphoric utterances are processed by the bilingual brain as well as how scientific context might modulate such processes. The present study aims to investigate the electrophysiological correlates of scientific metaphor comprehension in intermediate Chinese-English bilingual speakers. Our predictions are as follows. In the N400 window, due to the complicated contextual structure, scientific metaphors, either English or Chinese, might elicit higher N400 reflecting higher cognitive load in retrieving the stored information for meaning integration. More importantly, there should be N400 differences when processing Chinese (L1) and English (L2) scientific metaphors due to the fact that the lexical-semantic connection of Chinese scientific metaphors is stronger than that of English ones for the native Chinese speakers. English scientific metaphor processing should show higher amplitude, longer latency, and right-biased activation when compared with Chinese ones.

In the LPC window, scientific metaphors might elicit more negative late negativity for further integration of meaning to achieve the later knowledge-understanding inference. Moreover, there should be different patterns of late components when processing Chinese (L1) and English (L2) scientific metaphors due to the fact that the L2 structure especially involving complicated reasoning tends to be processed in a different way with the L1 structure. Chinese scientific metaphors with looser semantic relations might elicit greater right hemisphere activity compared with literal expressions. However, it should be very difficult for deep-level reintegration of meaning to occur in L2. Thus, compared with Chinese scientific metaphor processing, English scientific metaphor processing should show lower amplitude, shorter latency, and left-biased activation.

## Experiment 1: Comprehension of scientific metaphors in native language (Chinese)

Experiment 1 was set up as a control experiment to examine whether subjects showed the same pattern when processing scientific metaphors in L2 as in L1.

### Participants

All participants were undergraduate students at Shaanxi Normal University who were paid for participation. Twenty-three (10 males, 13 females, average age 20.6) right-handed subjects spoke Chinese as their native language and started to learn English from elementary period. They have passed CET-4 but have not passed CET-6 (CET is a national test of English for non-English majors in China), which means their proficiency of English is not high. Past or present mental or neurological disorders or major head injury, or crime are exclusion criteria. Handedness was tested using the Edinburgh Handedness Scale (Oldfield, 1971). The resulting average score was above 40 (*M* = 120.71, SD = 36.67), which means all participants were right-handed (left-handedness: lower than-40; ambidexter: between-40 and 40). The local Human Participation Research Review Board approved the experimental criteria for the study. Prior to participation, each subject provided written informed consent. However, due to a lack of available trials (lower than 85% of the total 80 trials based on artifact detection), data from six subjects were not included in the analysis, resulting in a final sample size of 17 subjects (eight males and nine females).

### Stimuli

For experiment 1, the materials were from our previous study (Tang et al., 2017a), in which several pretests had been done on familiarity, metaphoricity, and meaningfulness (see Table 1).

**Table 1:** The results of pretests.

	Meaningfulness		Figurativeness		Familiarity	
	*M*	SD	*M*	SD	*M*	SD
SM	3.5	0.6	3.3	0.24	2.85	0.59
LT	4.09	0.44	1.4	0.19	4.12	0.24

The stimulus pool consisted of 80 sentences, all in Chinese (see Table 2 for examples), which fell into two categories: scientific metaphoric (SM) and daily literal (LT), with 40 sentences in each sentence category. Grammar and syntactic structures were matched across different categories by adopting the “X 是 (shi) Y” format in all sentences and the lengths of the target words were balanced between categories, thus excluding possible confounding factors such as sentence length or complex syntactic processing. The frequencies of the target words were tested through BCC Corpus (Xun et al., 2016). A paired sample t-test showed that there was no significant difference (*t* = −1.93, *p* = 0.061) of the frequencies between the target words of Chinese scientific metaphors (*M* = 34032.9, SD = 7032.32) and those of Chinese literal expressions (*M* = 72092.1, SD = 19377.3). In order to control the concreteness of the target words across categories, only words considered as concrete were selected as stimuli and 40 raters with similar educational backgrounds, English levels and ages to the participants were asked to judge whether each target word is concrete or not on a 1–7 scale (1 = highly abstract, 7 = highly concrete). A paired sample t-test showed that there was no significant difference (*t* = −0.46, *p* = 0.648) of the concreteness between the target words of scientific metaphors (*M* = 5.3, SD = 0.22) and those of literal expressions (*M* = 5.36, SD = 0.28). Several pretests of meaningfulness, figurativeness and familiarity were conducted in our previous study.

**Table 2:** Chinese sample stimuli.

Scientific metaphors	淋巴是警察。	lin ba shi jing cha	Lymph is police.
	电子是行星。	dian zi shi xing xing	Electrons are planets.
	导体是隧道。	dao ti shi sui dao	Conductors are tunnels.
	染色体是姐妹。	ran se ti shi jie mei	Chromosomes are sisters.
	病毒是杀手。	bin du shi sha shou	Virus is killer.
Literal expressions	教授是学者。	jiao shou shi xue zhe	A professor is a scholar.
	汉语是语言。	han yu shi yu yan	Chinese is a language.
	伦敦是城市。	lundun shi cheng shi	London is a city.
	蚂蚁是昆虫。	ma yi shi kun chong	An ant is an insect.
	小狗是宠物。	xiao gou shi chong wu	The dog is a pet.

### Procedure

**Figure 1:**
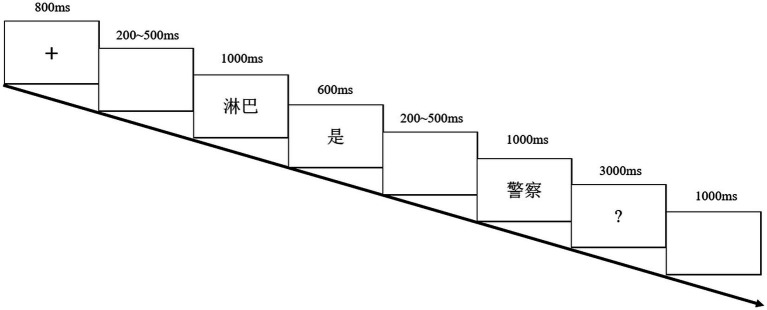
Experimental paradigm of experiment 1.

The experiment took place in a sound-attenuated, electrically shielded room. The sentences were presented in white color on a black background word by word in a quasi-random order. Stimuli on each trial were presented in the following time sequence: fixation cross (800 ms), blank (200–500 ms), subject (1,000 ms), verb (600 ms), blank (200–500 ms), object (1,000 ms) and question mark (3,000 ms). At the sight of the question mark, participants gave their judgments about whether the sentence was metaphoric or not by pressing a corresponding key with the right and left index fingers. Response period was limited to 3 s and was followed by a 1 s intertrial interval. The overall sequence of events for a trial is illustrated in Figure 1. Before the ERP experiment, in order to make sure that the academic knowledge involved in scientific metaphors could be understood during the experiment, participants firstly were asked to read a list of scientific terms, from which scientific metaphors were created, together with their brief explanations. Before the main session of the experiment, there was a brief practice session to familiarize the participants with the experimental procedure.

### Electrophysiological recording

Scalp voltages were collected with the CURRY 7 system (Compumedics Neuroscan, Texas, United States) with 64 Ag/AgCl electrodes, monitored by the CURRY recorder software and connected to a SynAmp amplifier (Compumedics Neuroscan, Texas, United States). Amplified analog voltages were digitized at 1,000 Hz. Impedances of individual sensors were kept below 5 kΩ. Eye movements were monitored through bipolar electrodes which were placed above and below the right eye, as well as at the left and right canthi. EEG was measured online with reference to the left mastoid, with a ground electrode on the medial frontal aspect, and later was analyzed offline with re-reference to an average of the left and right mastoids.

EEG was analyzed with the SCAN 4.5 software (Compumedics Neuroscan, Texas, United States) and Matlab using the ERPLAB toolbox (Lopez-Calderon and Luck, 2014). The EEG was digitally filtered at 0.1–30 Hz bandpass. Eye movements were corrected with an ocular artifact correction algorithm (Gratton et al., 1983). Artifacts with amplitudes exceeding ±75 μV were removed from analyses. ERPs were time-locked to the onset of the last word of the sentence and were obtained by stimulus-locked averaging of the EEG recorded in each condition. Epochs were 1,000 ms long with a 200 ms pre-stimulus baseline and the epochs baseline corrected using the-200–0 ms time window. The time windows of N400 (300–500 ms) and LPC (550–800 ms) were selected based on the Grand average ERP waveforms. Within these time intervals, the mean amplitude values of N400 and LPC as well as the 25% fractional area latency of N400 were determined. The resulting amplitudes of N400 and LPC as well as N400 latency values were entered into repeated measures analyses of variance (ANOVA). All ANOVA results were Greenhouse–Geisser corrected if assumption of sphericity was violated.

Event-related brain potentials were time-locked to the onset of the last word of the sentence. The resulting amplitudes of N400 were entered into 2 type (scientific metaphors, literal expressions) × 3 region (frontal F3, Fz, F4, central C3, Cz, C4, parietal P3, Pz, P4) × 3 hemisphere (left F3, C3, P3, midline Fz, Cz, Pz, right F4, C4, P4) three-way ANOVAs for repeated measures.

### Results

#### Behavioral performance

To achieve the target of this analysis, we calculated the mean response time for correct trials and the accuracy rate for each sentence type for each participant. A paired samples t-test revealed significant effects of type for both reaction times (*t* = 3.7, *p* = 0.002) and accuracy rates (*t* = −3.66, *p* = 0.002). Reaction times were longer for scientific metaphors (*M* = 520.11 ms, SD = 216.94 ms) than for literal expressions (*M* = 459.55 ms, SD = 186.75 ms). Accuracy rates were significantly lower for scientific metaphors (*M* = 0.83, SD = 0.087) than for literal expressions (*M* = 0.95, SD = 0.069).

#### Electrophysiological data

**Figure 2:**
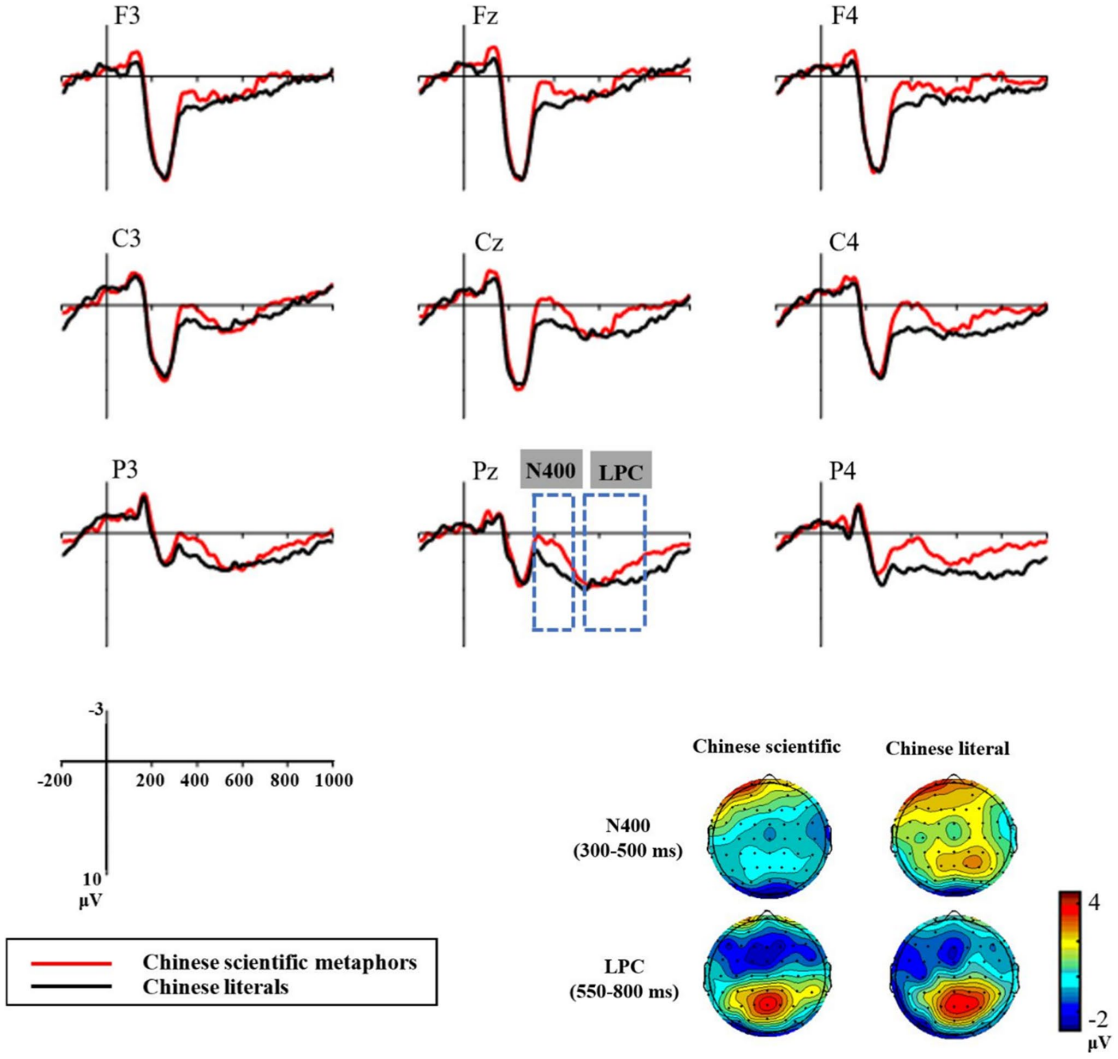
Grand average event-related potential (ERP) waveforms recorded at the 9 chosen electrodes of Chinese pairs.

In the time window of N400 (300 ~ 500 ms), the type (Chinese scientific metaphors, Chinese literal expressions) × region × hemisphere ANOVA revealed a significant main effect of type for amplitudes elicited by Chinese pairs [*F* (1, 16) = 103.2, *p* < 0.001, *η^2^_p_* = 0.87]. Scientific metaphors elicited more negative N400 (*M* = 0.55 μV, *SD* = 3.37 μV) than literals (*M* = 3.42 μV, SD = 3.2 μV; see Figure 2). For the N400 latency values, a similar repeated measures ANOVA revealed no significant main effect of type (*p* = 0.381).

In the time window of LPC (550 ~ 800 ms), the type × region × hemisphere ANOVA revealed a significant main effect of type for Chinese pairs [*F* (1, 16) = 9.09, *p* = 0.008, *η^2^_p_* = 0.36]. Consistent with Tang et al. (2017a), as shown in Figure 2, the ERPs of scientific metaphors (*M* = 1.53 μV, SD = 3.94 μV) were less positive than those of literal sentences (*M* = 3.15 μV, SD = 4.38 μV). There were significant type × region interactions [*F* (2, 32) = 5.55, *p* = 0.014, *η^2^_p_* = 0.26]. Simple effect tests showed significant differences between scientific metaphors and literal sentences in the parietal and central regions [Parietal: *F* (1, 16) = 11.37, *p* = 0.004, *η^2^_p_* = 0.42; Central: *F* (1, 16) = 5.8, *p* = 0.028, *η^2^_p_* = 0.27] but not in the frontal region (*p* = 0.42; See Figure 2).

### Discussion

The two main findings regarding N400 for the contextual factor and the late negativity for the knowledge-understanding inference factor are consistent with the findings of other studies (Tang et al., 2017a,b). From the conceptual blending view (Coulson and Van Petten, 2002; Yang et al., 2013), during the semantic processing to integrate these elements in metaphor comprehension, the amplitudes of N400 indicate the degree of difficulties in retrieving the stored conceptual knowledge. Consistent with previous studies (Rutter et al., 2012; Lai and Curran, 2013; Schneider et al., 2014), this study found that scientific metaphors elicited more negative N400s relative to literal expressions. Another possible explanation for these effects is the different salience between scientific metaphors and literal expressions. According to the graded salience hypothesis (Giora, 2003), the metaphoric meanings of scientific metaphors are less salient than the literal meanings which results in a more cognitive costly processing for meaning retrieving than literal expressions. In addition, consist with our previous study which reported higher N400 elicited by Chinese scientific metaphorical and literal expressions compared with Chinese daily literal expressions (Tang et al., 2016), the electrophysiological differences of N400s between Chinese scientific metaphors and Chinese literal expressions in present study might be related not only to metaphoric factor but also to scientific factor reflecting the modulation of complex scientific context of scientific metaphors.

Consistent with previous studies (Jankowiak et al., 2017, 2021; Tang et al., 2017a,b), scientific metaphors might elicit a more negative component partly overlapping in space and time with the LPC reducing the amplitudes of LPC. Such a late negativity might reflect secondary semantic integration processes of novel metaphors (Arzouan et al., 2007a,b; Goldstein et al., 2012; Rutter et al., 2012), showing a sustained reinterpretation process after an initial failure to reach meaning (Jiang et al., 2009) supporting the structural-mapping model (Gentner, 1983) and the career of a metaphor model (Gentner and Wolff, 1997; Bowdle and Gentner, 2005). Moreover, such electrophysiological differences of late negativity might be related to scientific factor consist with our previous study reflecting higher cognitive cost caused by the late knowledge-understanding inference of scientific language (Tang et al., 2016).

## Experiment 2: Comprehension of scientific metaphors in non-native language (English)

### Participants

The participants of Experiment 2 were the same as those of Experiment 1. In order to avoid the learning effects, experiment 2 was conducted 1 month later.

### Stimuli

The 80 English sentences form the stimulus pool and these sentences are the counterparts of the Chinese stimuli (see Table 3 for examples), which fell into two categories: literal and scientific metaphoric, with 40 sentences in each sentence category (Table 4). Similar to the pilot surveys we have done on the Chinese stimuli in our previous study used in Experiment 1 (Tang et al., 2017a), the English stimuli were tested by several groups of raters who did not participate in the ERP experiment. Only participants who had majored in a scientific discipline were selected so that they could avoid difficulties in understanding the scientific terms associated with the scientific metaphor categories (Table 4).

**Table 3:** English sample stimuli.

Scientific metaphors	Lymph is police.
	Electrons are planets.
	Conductors are tunnels.
	Chromosomes are sisters.
	Virus is killer.
Literal expressions	A professor is a scholar.
	English is a language.
	London is a city.
	An ant is an insect.
	The dog is a pet.

**Table 4:** Experimental materials.

	English scientific metaphors	English literal expressions	Chinese scientific metaphors	Chinese literal expressions
1	A charge is flow.	A professor is a scholar.	电荷是水流。	教授是学者。
2	Lymph is police.	Chinese is a language.	淋巴是警察。	汉语是语言。
3	Conductors are tunnels.	A boss is a man.	导体是隧道。	老板是富人。
4	Chromosomes are sisters.	The Great Wall is a monument.	染色体是姐妹。	丝绸是面料。
5	Virus is killer.	Silk is fabric.	病毒是杀手。	长城是古迹。
6	Numbers are spouses.	Strawberries are fruits.	数字是配偶。	草莓是水果。
7	Sound is wave.	London is a city.	声音是波浪。	伦敦是城市。
8	A sequences is a queue.	Peking Opera is drama.	数列是排队。	京剧是戏剧。
9	Chemical bonds are springs.	Stamp collecting is a hobby.	化学键是弹簧。	地震是灾害。
10	Dendrites are antennae.	Michael is a writer.	树突是天线。	老舍是作家。
11	A capacitor is a container.	An ant is an insect.	电容是容器。	蚂蚁是昆虫。
12	Benzene is a snake.	Earthquakes are disasters.	苯环是小蛇。	集邮是爱好。
13	Functions are machines.	Buddhism is a religion.	函数是机器。	佛教是宗教。
14	The nucleus is the sun.	Mary is a student.	原子核是太阳。	小明是学生。
15	A carbon atom is a orange.	Running is exercise.	碳原子是桔子。	跑步是运动。
16	A cell is a factory.	Grandma is an old man.	细胞是工厂。	奶奶是老人。
17	Inertia is the bank.	Eggplant is a vegetable.	惯性是银行。	茄子是蔬菜。
18	Circuits are bridges.	Cooking is a chore.	电路是桥梁。	做饭是家务。
19	Gears are teeth.	Christmas is a festival.	线粒体是密码。	春节是节日。
20	A root sign is a hat.	The dog is a pet.	根号是帽子。	小狗是宠物。
21	The virus is a worm.	Ginseng is a medicinal material.	电子云是马蜂群。	人参是药材。
22	A motor is a squirrel cage.	Painting is art.	电机是鼠笼。	绘画是艺术。
23	Helium gas is a lazy man.	China is a country.	气体是懒虫。	中国是大国。
24	Triangles are containers.	Parents are workers.	三角形是容器。	父母是工人。
25	Atomic groups are collectives.	The teacher is a woman.	原子团是集体。	教师是职业。
26	Operations are traffic.	Mosquitoes are pests.	运算是交通。	蚊子是害虫。
27	An empty set is an empty classroom.	Rice is a staple food.	空集是空教室。	大米是主食。
28	The power is a cotton coat.	Sofas are furniture.	次方是棉衣。	沙发是家具。
29	Matter is the signal.	Dumplings are foods.	物质是信号。	饺子是美食。
30	The bolt is a fishtail.	Colds are diseases.	作用力是爸爸。	感冒是疾病。
31	Fractions are mother and child.	West Lake is a lake.	分数是母子。	西湖是湖泊。
32	A hydrogen atom is an apple.	Refrigerators are electronic equipments.	氢原子是苹果。	沙发是家具。
33	Chemical reaction is scuffle.	Air crashes are accidents.	反应是混战。	空难是事故。
34	The sign of absolute value is pants.	The Yangtze River is a river.	绝对值是裤子。	李白是诗人。
35	Electrons are planets.	Mosquitoes are pests.	电子是行星。	蚊子是害虫。
36	Glass is foam.	Coleridge is a poet.	玻璃是泡沫。	长江是河流。
37	Catalysts are stimulants.	Frank is a champion.	催化剂是兴奋剂。	林丹是冠军。
38	Energy is money.	New York is a city.	能量是钱币。	西安是城市。
39	Atoms are jujube cakes.	Trees are plants.	原子是枣糕。	武警是军人。
40	Collections are letterboxes.	Fingerprints are evidence.	集合是信箱。	指纹是证据。

Firstly, 40 raters were asked to decide whether each English sentence is meaningful or not on a 1–3 scale (1 = not meaningful, 2 = somewhat meaningful, 3 = highly meaningful). According to the results of the meaningfulness judgment, sentences rated by at least 75% of the judges as metaphorically/literally plausible (> 2.5) were selected as expressions with either a metaphoric or a literal meaning. Then those selected sentences were rated by another group of 40 raters on a 1–3 scale regarding their figurativeness (1 = not figurative, 2 = somewhat figurative, 3 = highly figurative). In the present study, literal expressions, i.e., expressions averaging less than 1.5, and scientific metaphors averaging greater than 2.5. Finally, we asked another group of 40 raters to rate these expressions on a 7-point familiarity scale ranging from 1 (highly unfamiliar) to 7 (highly familiar). Expressions scoring more than 4 on this scale were selected as scientific metaphors (mean rating 5.33) and literal expressions (mean rating 6.32).

The frequencies of the target words of those English stimuli were also tested through Brown Corpus. A paired sample t-test showed that there was no significant difference (*t* = −0.99, *p* = 0.33) of the frequencies between the target words of English scientific metaphors (*M* = 77.08 per million, SD = 25.9) and those of English literal expressions (*M* = 106.98 per million, SD = 28.45).

### Procedure

**Figure 3:**
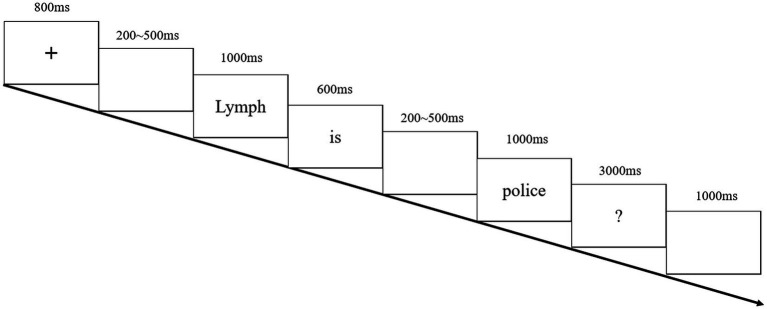
Experimental paradigm of experiment 2.

Experiment 2 was conducted following the same procedure of Experiment 1 but in English (see Figure 3).

### Electrophysiological recording

The electrophysiological recording of Experiment 2 was the same as that of Experiment 1.

### Results

#### Behavioral performance

For the purposes of this analysis, we calculated the mean response time for correct trials and the accuracy rate for each sentence type for each participant. A paired samples t-test revealed significant effects of type for accuracy rates (*t* = −4.89, *p* < 0.001). Accuracy rates were significantly lower for English scientific metaphors (*M* = 0.84, SD = 0.07) than for English literal expressions (*M* = 0.85, SD = 0.07). The difference in reaction times between scientific metaphors and literal expressions was not found to be significant (*p* = 0.471, SM: *M* = 591.04 ms, SD = 176.21 ms; LT: *M* = 591.59 ms, SD = 177.64 ms).

#### Electrophysiological data

##### 300 ~ 500 ms

**Figure 4:**
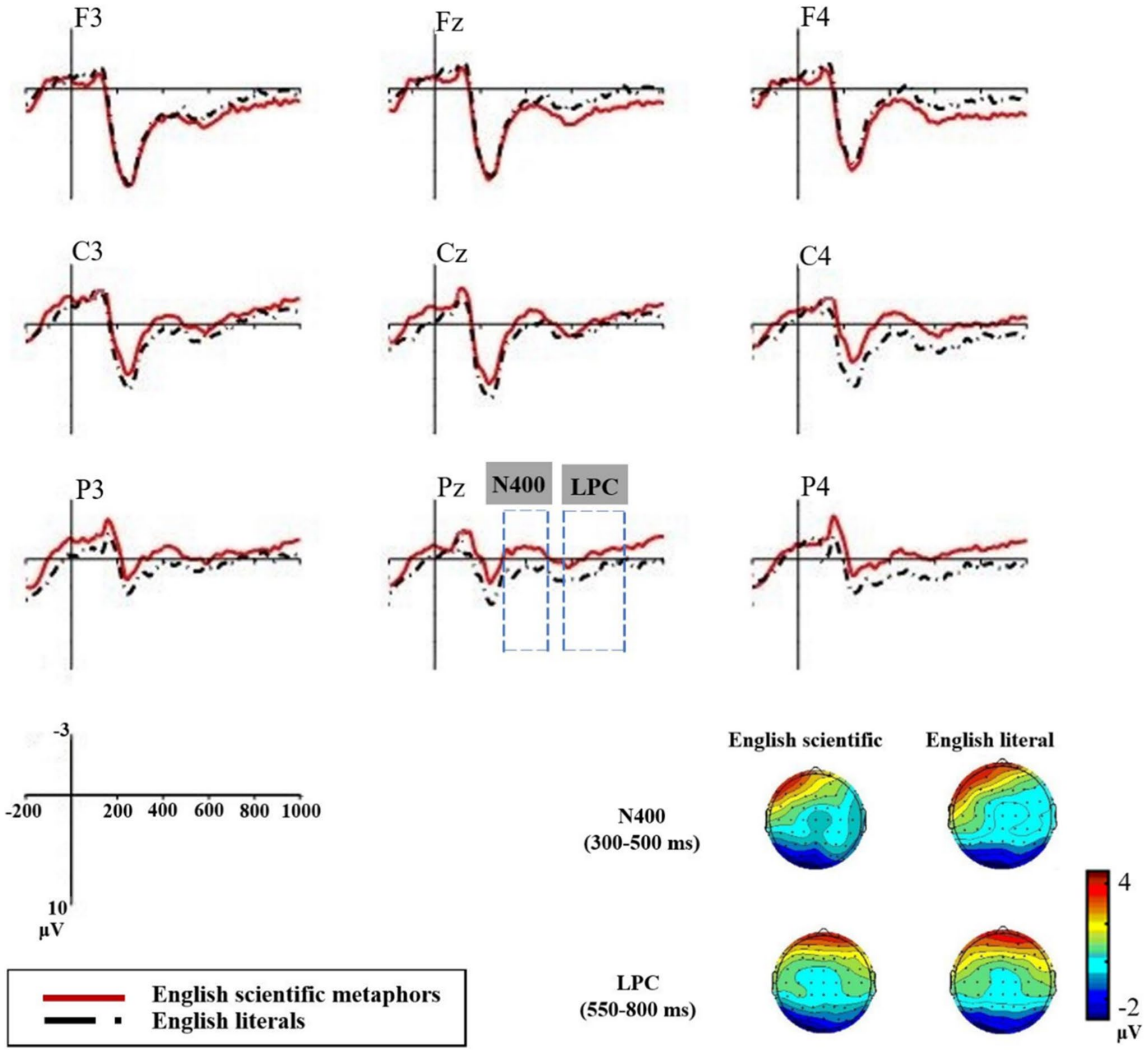
Grand average ERP waveforms recorded at the nine chosen electrodes of English pairs.

As shown in Figure 4, in the time window of N400 (300 ~ 500 ms), there were significant main effects of type (English scientific metaphors, English literal expressions) [*F* (1, 16) = 62.26, *p* < 0.001, *η^2^_p_* = 0.79]. Scientific metaphors elicited more negative N400 (*M* = −1.35 μV, SD = 3.01 μV) than literal sentences (*M* = 1.5 μV, SD = 2.59 μV). For the N400 latency values, similar to the result of experiment 1, no significant main effect of type (*p* = 0.288) was found.

##### 550 ~ 800 ms

###### LPC

In the time window of LPC (550 ~ 800 ms), there were significant main effects of type [*F* (1, 16) = 57.63, *p* < 0.001, *η^2^_p_* = 0.78]. As shown in Figure 4, the ERPs of scientific metaphors (*M* = −1.43 μV, SD = 3.08 μV) were less positive than those of literal sentences (*M* = 0.98 μV, SD = 2.85 μV). There were significant type × region interactions [*F* (2, 32) = 13.89, *p* < 0.001, *η^2^_p_* = 0.47]. Simple effect tests showed great significant differences between English scientific metaphors and literal sentences in the parietal and central regions [Parietal: *F* (1, 16) = 61.73, *p* < 0.001, *η^2^_p_* = 0.79; Central: *F* (1, 16) = 76.51, *p* < 0.001, *η^2^_p_* = 0.83] (see Figure 4).

### Discussion

Similar to the Chinese pairs, English (L2) scientific metaphors also elicited more negative N400 than literal ones reflecting more demanding retrieving of stored conceptual knowledge due to the mapping between the scientific target and the daily source. Two aspects could be explained this discrepancy: either English scientific metaphors are difficult to process because of the need to reject literal meanings and retrieve appropriate metaphorical meanings (Wang and Jankowiak, 2021), or bilinguals have difficulty transferring explicit L2 knowledge to their implicit language skills (Chen et al., 2013). Furthermore, English scientific metaphors also elicited more negative late negativity reflecting the secondary integration of meaning due to the late analogical comparison involved in processing scientific metaphors.

## Comparative analysis between L1 and L2

### Results

#### Behavioral performance

A further language (Chinese, English) × type (scientific metaphor, literal expression) repeated-measures ANOVA revealed significant effects of language for accuracy rates [*F* (1, 16) = 4.81, *p* = 0.043, *η^2^_p_* = 0.12]. The accuracy rates were significantly lower for English expressions (*M* = 0.85, SD = 0.02) than those for Chinese expressions (*M = 0.*89, SD *=* 0.01). Moreover, a repeated-measures ANOVA revealed significant effects of language for reaction times [*F* (1, 16) = 4.66, *p* = 0.046, *η^2^_p_* = 0.23]. The reaction times of English expressions (*M* = 591.32 ms, SD = 42.91 ms) were significantly longer than those of Chinese expressions (*M* = 489.83 ms, SD = 48.4 ms).

#### Electrophysiological data

##### 300 ~ 500 ms

###### N400

**Figure 5:**
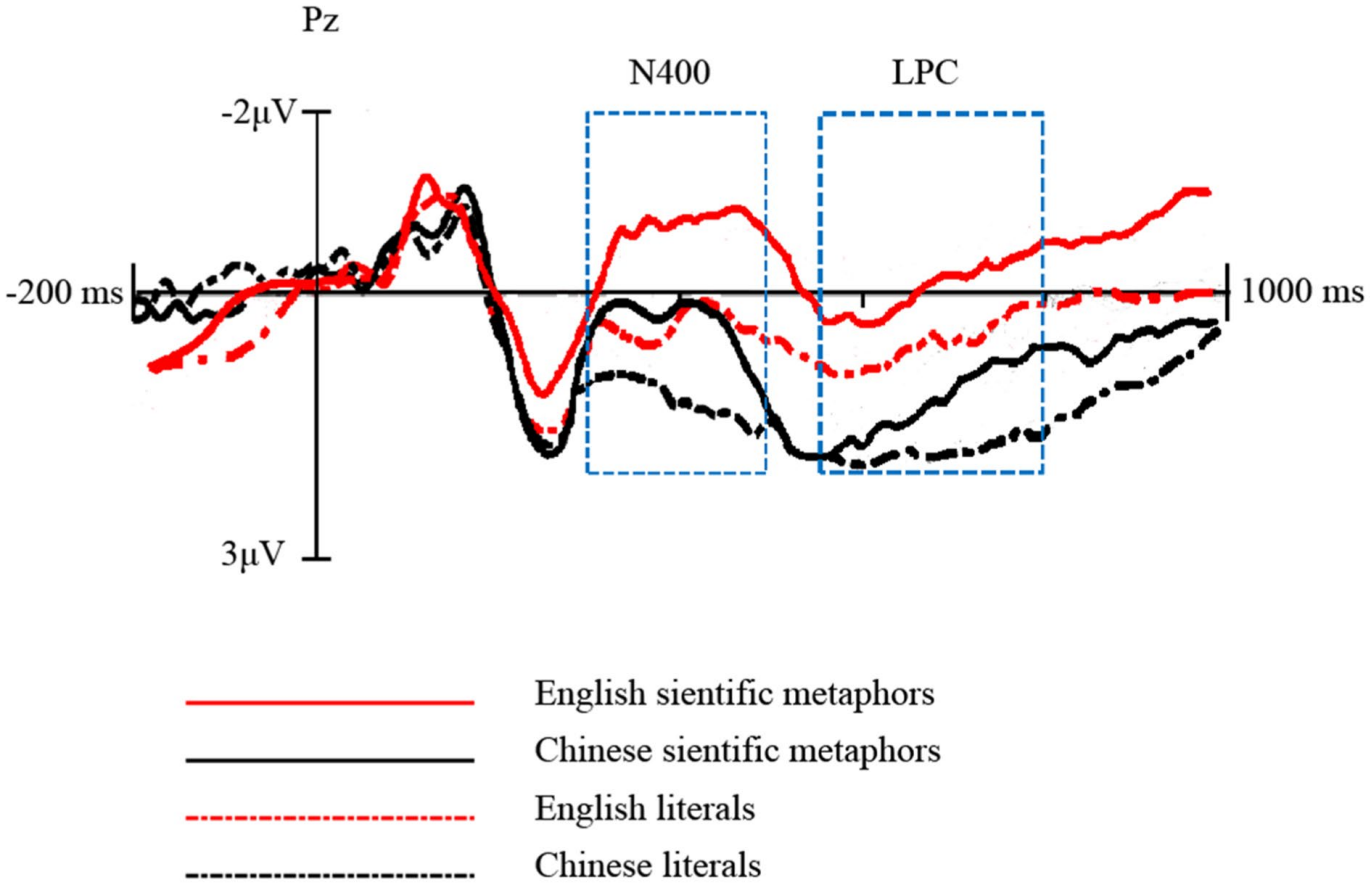
Grand average ERP waveforms recorded at the Pz electrodes of four conditions

A language (Chinese, English) × type (scientific metaphors, literal expressions) × region × hemisphere repeated-measures ANOVA was performed for the N400 amplitudes of English and Chinese scientific metaphoric pairs and literal pairs. As shown in Figure 5, the main effects of language were salient [SM: *F* (1, 16) = 6.47, *p* = 0.022, *η^2^_p_* = 0.29; LT: *F* (1, 16) = 8.39, *p* = 0.011, *η^2^_p_* = 0.34], with English stimuli eliciting more negative N400 than Chinese stimuli [English stimuli: *M* = 0.08 μV, SD = 0.43 μV; Chinese stimuli: *M* = 1.99 μV, SD = 0.61 μV].

Significant language × region interactions were found in both pairs [SM: *F* (2, 32) = 6.47, *p* = 0.022, *η^2^_p_* = 0.29; LT: *F* (2, 32) = 8.49, *p* = 0.011, *η^2^_p_* = 0.34]. Simple effect tests showed that English sentences elicited more negative N400 in the central and parietal regions (*ps* < 0.05). Language × hemisphere interactions were not significant for the scientific metaphoric pairs (*p* = 0.14), but marginally significant for the literal pairs [*F* (2, 32) = 3.33, *p* = 0.064, *η^2^_p_* = 0.17]. Simple effect tests showed that English and Chinese literals differed significantly in the midline and the right hemisphere (*ps* < 0.05). The results indicate that both hemispheres play a significant role in the comprehension of English sentences by Chinese-English bilinguals, with the right parietal area of the right hemisphere necessarily involved.

For the N400 latency values, a similar repeated-measure ANOVA revealed no significant language effect for both scientific metaphoric pairs and literal pairs (SM: *p* = 0.145, LT: *p* = 0.978). Language × region interactions were not significant (*p =* 0.408). However, there were significant language × hemisphere interactions [*F* (2, 32) = 4.79, *p* = 0.018, *η^2^_p_* = 0.23]. Simple effects tests showed that the mean N400 latency of English stimuli (*M* = 380.45 ms, SD = 1.34 ms) was marginally significantly later than that of Chinese stimuli (*M* = 376.35 ms, SD = 1.76 ms) in the midline (*p* = 0.073). Moreover, language × type × hemisphere interactions were marginally significant [*F* (2, 32) = 3.46, *p* = 0.066, *η^2^_p_* = 0.18]. Simple effects tests showed that the N400 latencies of English scientific metaphors were significantly later than that of Chinese scientific metaphors in the midline and the right hemisphere (*ps* < 0.05) where no significant difference between N400 latencies of English and Chinese literal expressions was found.

##### 550 ~ 800 ms

###### LPC

In the time window of LPC (550 ~ 800 ms), for the LPC amplitudes of English and Chinese scientific metaphoric pairs and literal pairs, the main effects of language were both significant [SM: *F* (1, 16) = 12.18, *p* = 0.003, *η^2^_p_* = 0.43; LT: *F* (1, 16) = 4.54, *p* = 0.049, *η^2^_p_* = 0.22], with English stimuli eliciting less positive LPCs than Chinese stimuli [English stimuli: *M* = −0.23 μV, *SD* = 0.48 μV; Chinese stimuli: *M* = 2.34 μV, *SD* = 0.73 μV]. Significant language × region interactions were found in both pairs [SM: *F* (2, 32) = 14.34, *p* < 0.001, *η^2^_p_* = 0.47; LT: *F* (2, 32) = 13.21, *p* < 0.001, *η^2^_p_* = 0.45]. Simple effect tests showed that both English scientific metaphors and literals elicited less positive LPC in the parietal regions (*ps* < 0.01) and English scientific metaphors also elicited less positive LPC in the central region (*p* = 0.011). Language × hemisphere interactions were not significant for the two pairs (*ps* > 0.1).

###### The sustained late negativity

**Figure 6:**
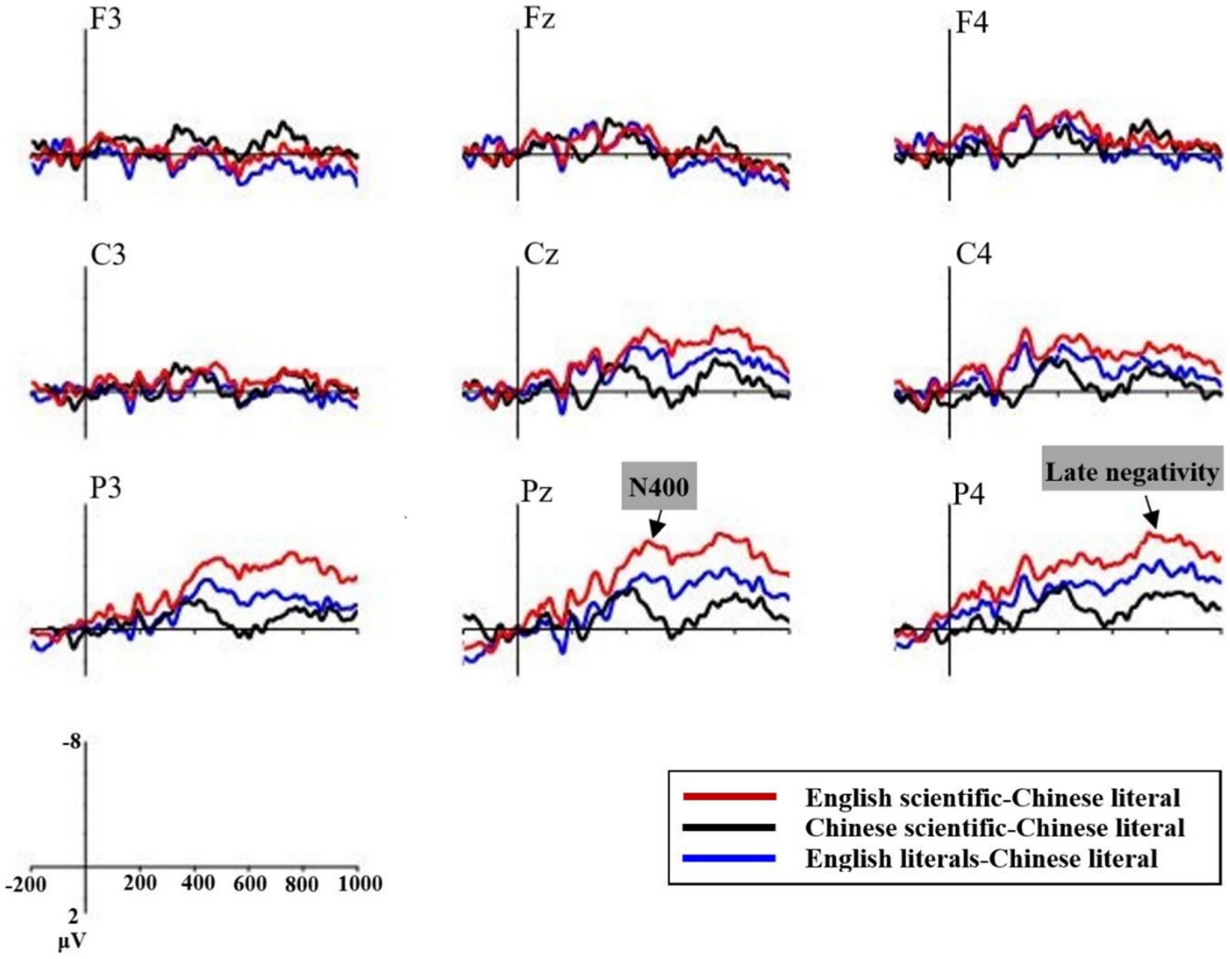
Grand average ERP waveforms recorded at the nine chosen electrodes of the late negativity

Moreover, the ERP patterns in the late window (550–800 ms) were further illustrated by subtracting the amplitude of Chinese literal expressions from those of English scientific metaphoric, English literal and Chinese scientific metaphoric expressions respectively, and the resulting difference curves are shown in Figure 6. Following their prominent N400 effects, the two English conditions and Chinese scientific metaphors displayed a second negativity peaking around 760 ms. There were significant main effects of type [*F* (2, 32) = 9.25, *p* = 0.005, *η*^2^*_p_* = 0.37]. The two English conditions elicited more negative amplitudes than Chinese scientific metaphors (ESM: *M* = −4.58 μV, SD = 5.89 μV; ELT: *M* = −2.17 μV, SD = 5.84 μV; CSM: *M* = −1.62 μV, SD = 3.74 μV; E refers to English while C refers to Chinese). The interaction effects between type and region [*F* (4, 64) = 11.09, *p* < 0.001, *η*^2^*_p_* = 0.41] indicated that the differences were more prominent at the central and parietal regions.

For the English pairs, there were main effects of type [*F* (1, 16) = 57.63, *p* < 0.001, *η*^2^*_p_* = 0.78]. There were significant type × region interactions [*F* (2, 32) = 13.89, *p* < 0.001, *η*^2^*_p_* = 0.47]. Simple effect tests showed significant differences in all the three regions [*Fs* (1, 16) > 15, *ps* < 0.01] with the more significant differences existing in the central and parietal regions [*Fs* (1, 16) > 60, *ps* < 0.001].

For English and Chinese scientific metaphoric pairs, there were main effects of language [*F* (1, 16) = 12.18, *p* = 0.003, *η*^2^*_p_* = 0.43]. Language × region interactions were significant [*F* (2, 32) = 14.34, *p* < 0.001, *η*^2^*_p_* = 0.47]. Simple effect tests showed significant differences in the parietal region [*F* (1, 16) = 37.95, *p* < 0.001, *η*^2^*_p_* = 0.7] and the central region [*F* (1, 16) = 8.26, *p* = 0.011, *η*^2^*_p_* = 0.34], but not in the frontal region (*p* = 0.41).

For English literal and Chinese scientific metaphoric pairs, type × region interaction was significant [*F* (2, 32) = 7, *p* = 0.004, *η*^2^*_p_* = 0.31] with the significant difference existing in the parietal region [*F* (1, 16) = 7.24, *p* = 0.016, *η*^2^*_p_* = 0.31], but not in the frontal and central regions (*ps* > 0.9). In order to make a clear comparison between the two conditions, separate pairwise ANOVAs for all the three electrodes in the parietal region were performed. The differences were very significant at the right site [P4: *F* (1, 16) = 16.72, *p* = 0.001, *η*^2^*_p_* = 0.51], significant at the central site [Pz: *F* (1, 16) = 5.79, *p* = 0.029, *η*^2^*_p_* = 0.27], and not significant at the left site (P3: *p* = 0.16).

### Discussion

#### The different familiarities of L1 and L2

Firstly, in this study, English literals elicited more negative N400 than Chinese literals. The N400 indexes the difficulty in retrieving the stored information of a word (Coulson and Van Petten, 2002; Yang et al., 2013). Greater N400 amplitudes for L2 daily literal expressions might result from their unfamiliarity (Proverbio et al., 2002; Moreno et al., 2008; Midgley et al., 2009; Newman et al., 2012; Heidlmayr et al., 2015). For late learners, L1 daily words are used very frequently in people’s life but it is not mostly the case for L2 daily words. Naturally, exposure to L1 words is much higher than exposure to L2 words, resulting in the lower familiarity of L2 words. Therefore, processing the meaning of L2 words appears more difficult than that of L1 words (Wang and Chen, 2020).

Secondly, it is known from previous studies of N400 in bilingual semantic processing (WeberFox and Neville, 1996; Phillips et al., 2004; Moreno and Kutas, 2005; Braunstein et al., 2012) that N400 latencies are longer for English words than for Chinese words. The present study also reported longer N400 latencies of English stimuli compared with that of Chinese stimuli in the midline, which was also consistent with the behavioral results showing reaction times of English stimuli were longer than that of Chinese stimuli. As can be seen from the BIA+ model (Dijkstra and van Heuven, 2002) the time delay assumed by the lower subjective frequency of L2 items may lead to delayed activation of semantic representations in non-native speakers. Thus, automatic operations involved in lexical-semantic access are less frequent when dealing with non-dominant languages with lower resting level activation (Jankowiak et al., 2017). Besides, L2 is acquired in a fundamentally different way from L1, so the lexical-semantic connection of L2 is weaker than that of L1 (Midgley et al., 2009; Palmer et al., 2010). L2 is mostly acquired explicitly during formal classroom instruction at school age, whereas native languages are always acquired implicitly during childhood.

#### The N400 regarding the scientific contextual factor

Studies have shown that the N400 observed for metaphor is related to context and may be an index of contextual expectations for upcoming words, guiding lexical access and retrieval (Bambini et al., 2016). In summary, scientific metaphors are more contextually complex than traditional metaphors, as they cover both scientific target domains and everyday source domains. It is clear from the results of this study that the N400 model of L1 and L2 processing of scientific metaphors is different.

Firstly, in this study, consistent with our predictions, English scientific metaphoric elicited more negative N400 than Chinese ones. The words in the source domains of either English or Chinese scientific metaphors were from daily life. As mentioned in the above discussion, retrieving the literal meaning of L2 words should be more difficult due to their lower familiarity and frequencies of usage. More importantly, the words used in the target domains of either English or Chinese scientific metaphors were scientific terms. Compared with daily words, exposure to scientific terms should be lower. Compared with L1 scientific terms, late learners’ exposure to L2 scientific terms should be even lower. Therefore, the different degrees of difficulties in processing L1 and L2 words is shown more clearly by adopting scientific terms in this study.

Secondly, following the retrieval of literal meaning of words in the daily source and scientific target domains respectively, processing scientific metaphors involves the integration of the two heterogeneous domains. Another possible reason for greater N400 amplitudes for L2 scientific metaphors lies in their complicated contextual structure, further enhancing the difficulty in integrating meaning. Some studies have shown that the integration of the two domains of L2 conventional metaphors costs more energy because the lexical-semantic connection between L2 words are weaker than that between L1 words (Midgley et al., 2009). Other studies have shown that the integration of the two domains of L1 scientific metaphors is more difficult than that of L1 conventional metaphors due to the longer distance between the two heterogeneous domains of scientific ones than that between those of conventional ones (Tang et al., 2017a,b). Based on the two findings, especially for late L2 learners, integrating the two domains of L2 scientific metaphors should be much more demanding due to the context of weaker lexical-semantic connection of words and longer distance of mapping.

Thirdly, consistent with our predictions, the mean N400 latency of English stimuli was longer than that of Chinese ones in the midline. As mentioned above, recognizing the literal meaning of L2 words, especially scientific terms, takes longer time (Taft et al., 2021). Moreover, the N400 latencies of English scientific metaphors were significantly longer than that of Chinese scientific metaphors in the midline and the right hemisphere revealed the unique time-course processing of scientific metaphors compared with literal expressions. Unlike the difference between L1 and L2 daily words, the scientific terms of both L1 and L2 are mostly learned explicitly with the method of formal classroom teaching at school age. Generally, scientific terms are used in some academic context, where L1 is often used to express difficult and abstract ideas and knowledge. According to the inhibitory control (IC) model (Green, 1998), in the L2 (English) context, it is difficult to inhibit L1 representation for scientific metaphors. The predominantly used non-dominant language may largely incur switching costs (Kalinka et al., 2018).

Moreover, the semantic connection between scientific terms and daily words should be weaker than that between different daily words. Therefore, compared with L1 scientific terms, the semantic connection of L2 scientific terms should be even lower. Hence the mapping of L2 scientific metaphors in the context of two heterogeneous domains might take more time (Jankowiak et al., 2017).

#### The late component regarding The reasoning factor

Some studies have shown that traditional and poetic metaphors have emotionally stimulating functions, while scientific metaphors have unique properties of knowledge comprehension, involving late reasoning processes (Tang et al., 2017a,b). The findings of the current research indicate that the late component patterns are much more complicated for L2 processing.

In this study, both English conditions (scientific metaphoric and literal) elicited less positive LPC than their Chinese counterparts. The reason might be that in the late time window when structural integration occurs semantic integration is prolonged (Kaan et al., 2000; Friederici, 2002). Firstly, the LPC has been thought to reflect the level of syntactic processing. The higher amplitudes of LPC for L1 might indicate that processing L1 involves more syntactic analysis while processing L2 mostly involves semantic analysis (Kotz, 2009). Secondly, from the perspective of semantic integration, the enhanced LPC for L1 might show the deeper processing of knowledge-reasoning of L1 scientific metaphors (Jankowiak et al., 2017).

Moreover, a late negativity was elicited in the time window of LPC. The late negativity has been found and reported by some monolingual (Arzouan et al., 2007a,b; Goldstein et al., 2012; Rutter et al., 2012) and bilingual studies on metaphor comprehension (Jankowiak et al., 2017; Jankowiak et al., 2021; Wang and Jankowiak, 2021), marking the ongoing difficulty of the secondary semantic integration especially of novel metaphors (Tang et al., 2017a,b) or the activated non-literal routes when understanding complex semantics in non-native contexts (Jankowiak et al., 2017).

In this study, inconsistent with our predictions, the two English conditions elicited more negative amplitudes than Chinese scientific metaphors. Firstly, the enhanced late negativity of English scientific metaphors might show that comprehending L2 scientific metaphors is more difficult, since the late negativity may mark the ongoing difficulty of integrating the two concepts. However, generally speaking, the depth of semantic integration for L2 metaphors could not reach the same magnitude as that for L1 metaphors (Midgley et al., 2009), especially when the late complicated reasoning process is involved in understanding the abstract concept related with scientific metaphors, which was also supported by the behavioral data showing that the difference of reaction times was not significant between English scientific metaphors and English literal expressions but significant between the two English conditions and Chinese scientific metaphors. Secondly, processing daily literals does not involve the late reasoning process for understanding related knowledge. Therefore, the late negativity with enhanced amplitudes observed for processing English conditions might probably imply a switching from L2 to L1 in the late period of processing L2, which elaborated the amplitudes of English conditions in this study. The late negativity elicited by the codes switch from L2 to L1 (from the weaker language to the dominant language) has been reported to suggest that switching may engage the activation costs of the specific lexical forms in the less active language and effortful sentence-level restructuring mechanisms (Palmer et al., 2010; Shukhan Ng et al., 2014; Litcofsky and Van Hell, 2017; Zeller, 2020).

#### The hemispheric involvement

Consistent with our predictions and previous studies (Jankowiak et al., 2017), during the earlier time window (N400), the processing of English (L2) expressions show a right-biased distribution of the brain. The lexical-semantic connection between L2 words is much weaker than that between L1 words (Midgley et al., 2009; Naranowicz et al., 2022), leading to a right-biased distribution. Based on fine-coarse semantic coding theory (Beeman, 2005), the coarse semantic encoding of the right hemisphere loosely activates and maintains a larger semantic domain, in which the semantic associations are more distant and meanings are more unconventional, while the fine semantic encoding of the left hemisphere focuses on a single dominant interpretation. Moreover, the significant difference of N400 latencies between English scientific metaphors and Chinese scientific metaphors in the midline and the right hemisphere also indicates a unique role of the right hemisphere for the processing of scientific metaphors. During the later time window (the late components), inconsistent with our predictions, the processing of English (L2) expressions show a right-biased distribution of the brain. However, probably coarse semantic coding might be much weaker for a non-native than native language (Miriam et al., 2012). That is to say, the coarse semantic processing might be too difficult for L2 learners so that they might switch to the fine semantic processing of L2. That is to say, the integration of two distant domains involving the scientific reasoning increases the difficulties of L2 processing so that L2 users might switch to their L1 in this case. Therefore, the right-biased distribution of the late negativity elicited by English scientific metaphors might result firstly from the code switch from English to Chinese, and secondly from the coarse semantic processing of scientific metaphors (Ng et al., 2014; Litcofsky and Van Hell, 2017).

## Conclusion

Overall, factors that interfere with the processing of metaphors include the different lexical encodings in the two languages of bilinguals (Xue et al., 2014). As demonstrated in the experiments reported here, the dynamics of processing literal and figurative meaning over time suggest a complex pattern, with language, context, inference and salience jointly modulating temporal dynamics and possible cerebral asymmetries, supporting the revised hierarchical model and the graded salience hypothesis. For processing L1 and L2 scientific metaphors, several factors interact complicatedly to determine their different ways throughout the course of semantic integration as well as the unique hemispheric involvement supporting the fine-coarse semantic coding theory.

Due to the unique properties of scientific metaphors, non-native speakers have difficulty experiencing deep integration of meaning. In addition, few studies have addressed the qualitative differences in non-literal representations in the brains of native and non-native speakers. The present study may be of help to give some sort of significant implications for the related further studies. In this important but surprisingly under-researched area, future research could be directed towards comparing the dynamics of processing scientific metaphors with other forms of metaphors such as conventional and poetic metaphors between native and non-native speakers. Meanwhile, the present study only investigated the electrophysiological differences between scientific metaphors and daily literal expressions which could be resulted by both metaphoric effect and scientific effect. Further research could explore the influence of those two effects independently by adding scientific literal expressions as stimuli to reveal the unique processing of scientific metaphors in the bilingual brain.

## Data availability statement

The raw data supporting the conclusions of this article will be made available by the authors, without undue reservation.

## Ethics statement

The studies involving human participants were reviewed and approved by Ethics Committee in Shaanxi Normal University. The patients/participants provided their written informed consent to participate in this study.

## Author contributions

XT contributed to conception and design of the study. XL, YH, and SH performed the data collection. LS, XT, XL, XG, ZY, and ZM performed the analysis. LS and XT wrote the first draft of the manuscript. All authors contributed to the article and approved the submitted version.

## Funding

The current research was funded by the National Social Science Foundation of China (Grant No. 17BYY092).

## Conflict of interest

The authors declare that the research was conducted in the absence of any commercial or financial relationships that could be construed as a potential conflict of interest.

## Publisher’s note

All claims expressed in this article are solely those of the authors and do not necessarily represent those of their affiliated organizations, or those of the publisher, the editors and the reviewers. Any product that may be evaluated in this article, or claim that may be made by its manufacturer, is not guaranteed or endorsed by the publisher.
